# Heterogeneous effects of COVID-19 control measures on tuberculosis in South Korea: an analysis of case notification data

**DOI:** 10.1186/s12931-022-01966-2

**Published:** 2022-03-11

**Authors:** Honghyok Kim, Young Ae Kang, Hee-Jin Kim, Hongjo Choi

**Affiliations:** 1 School of Environment, Yale University, New Haven, CT USA; 2 Division of Pulmonology and Critical Care Medicine, Department of Internal Medicine, Severance Hospital, Yonsei University College of Medicine, Seoul, Republic of Korea; 3 Korean National Tuberculosis Association, Seoul, Republic of Korea; 4 Department of Preventive Medicine, Konyang University College of Medicine, Daejeon, Republic of Korea

**Keywords:** COVID-19, Coronavirus disease, Tuberculosis, Social distancing, Health care

## Abstract

Coronavirus disease (COVID-19) responses such as social distancing practices can decrease health care access and tuberculosis (TB) notification, particularly among individuals aged 60 years or older. Conversely, they can increase TB notification among younger individuals. These results may be attributable to household transmission and the similarity of TB respiratory symptoms to COVID-19.

## Background

Several studies suggest that the disruption of health care services due to coronavirus disease (COVID-19) can delay tuberculosis (TB) diagnosis in the short term and lead to an elevated TB burden in the mid to long term [1, 2]. However, predicting the impact of the COVID-19 pandemic and control measures is not simple, and they may have a mixture of positive and negative effects on TB [3–5]. Social distancing and lockdowns curtail social activities and thus reduce the risk of the spread of *Mycobacterium tuberculosis* [5]. Conversely, lockdowns increase the frequency and intensity of contact among household members and thus can increase the risk for the spread of TB within households [3]. Similarly, the COVID-19 pandemic can have varying impacts on TB according to sociodemographic factors. The aim of our study was to determine the impact of social distancing during the COVID-19 pandemic on the TB case notification rate (CNR) according to age and sex.

## Methods

We analyzed the TB CNR trends in South Korea from 2012–2020 to investigate changes in the CNR during the COVID-19 pandemic. The TB case notification data from 2012–2020 was obtained from the Annual Report on the Notified Tuberculosis in South Korea published by the Korea Disease Control and Prevention Agency [6]. The annual report included only publicly available aggregated data. Data on the annual mid-year populations were obtained from the Korean Statistical Information Service. The crude TB CNRs from 2012–2020 were calculated based on the annual mid-year populations. The rates were calculated for all ages and by 10-year age groups (20–29 years, 30–39 years, …70–79 years), 0–19 years, and ≥ 80 years. The crude CNRs were analyzed by sex and previous TB history (new case or previously treated case) to determine the secular trend from 2012–2019. The estimated regression coefficient was used to project the crude CNR in 2020. The estimated 2020 crude CNR was compared with the observed 2020 crude CNR. All analyses were conducted using R software (version 3.5.3, http://cran.r-project.org; R Foundation for Statistical Computing, Vienna, Austria).

## Results

**Fig. 1:**
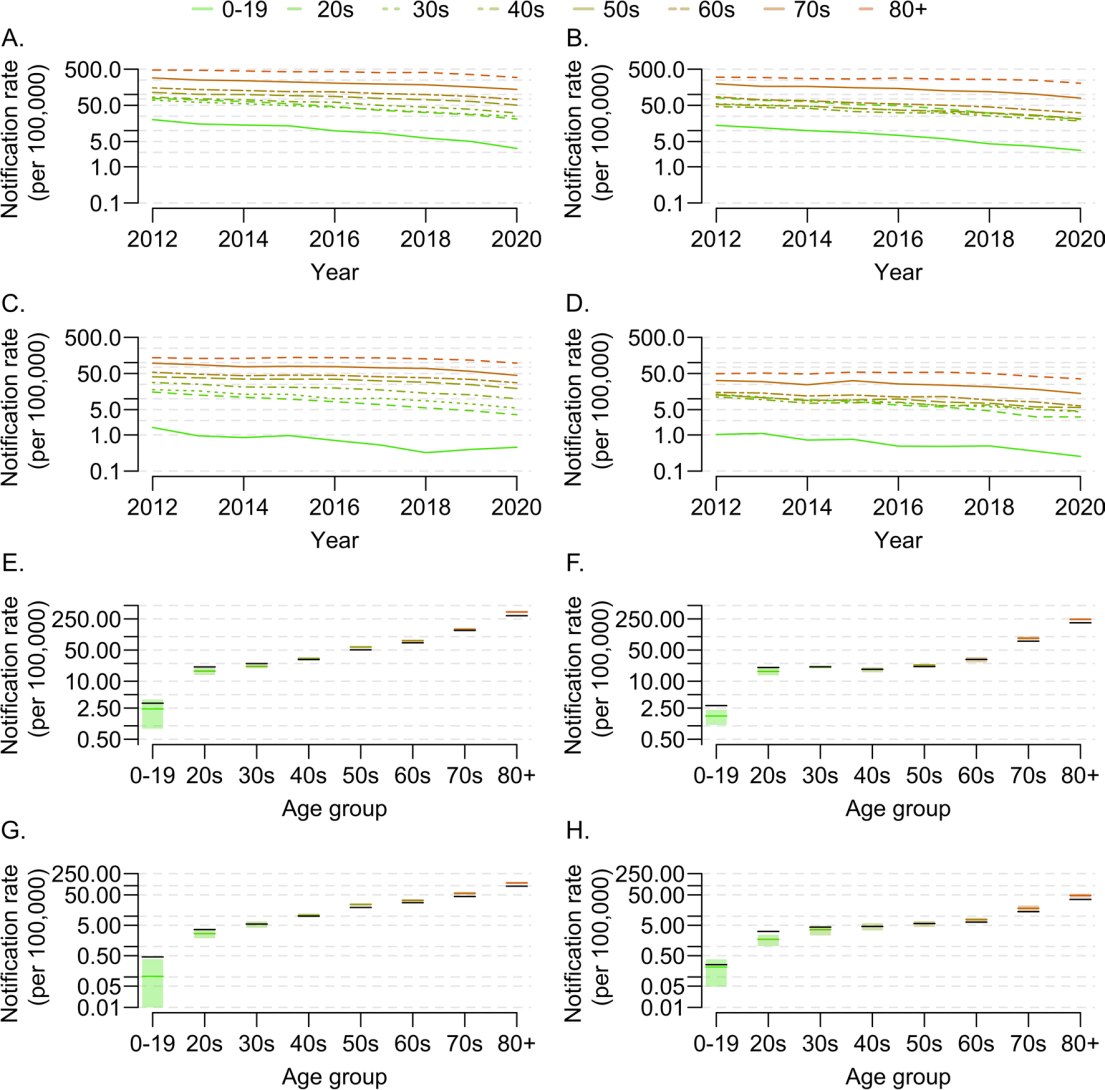
Observed time-trend for tuberculosis case notification rate in South Korea from 2012 to 2020 and the difference between observed and estimated tuberculosis case notification rates in 2020. **A** Observed time-trend for TB CNR for new cases in males by age groups. **B** Observed time-trend for TB CNR for new cases in females by age groups. **C** Observed time-trend for TB CNR for previously treated cases in males by age groups. **D** Observed time-trend for TB CNR for previously treated cases in females by age groups. **E** Observed value and estimated point estimate for TB CNR for new cases in males for the year of 2020. **F** Observed value and estimated point estimate for TB CNR for new cases in females for the year of 2020. **G** Observed value and estimated point estimate for TB CNR for previously treated cases in males for the year of 2020. **H** Observed value and estimated point estimate for TB CNR for previously treated cases in females for the year of 2020. In Figures **E**–**H**, the black line is the observed value; the colored line is the estimated point estimate; the colored bars show the 95% confidence intervals. *CNR* case notification rate, *TB* tuberculosis

The observed 2020 TB CNR in South Korea was the lowest since 2012 (49/100,000). The TB CNR decreased by 5–10% yearly between 2012–2019, and the CNR in 2020 was 16.4% lower than that in 2019 (Fig. 1A–D). When 2020 observed CNR was compared to 2020 estimated CNR based on the 2012–2019 CNR trends, the 2020 CNR was 10.2% lower than expected in males (crude CNR: 60/100,000 vs estimated CNR: 67/100,000) and 8.3% lower than expected in females (crude CNR: 39/100,000 vs estimated CNR: 42/100,000). However, the reduction of the 2020 TB CNR differed significantly according to age (Table 1).

**Table 1:** Difference between observed tuberculosis case notification rate and estimated tuberculosis case notification rate

Age group	New cases (male)		New cases (female)		Previously treated cases (male)		Previously treated cases (female)	
	Diff (95% CI)	P-value	Diff (95% CI)	P-value	Diff (95% CI)	P-value	Diff (95% CI)	P-value
0–19	0.8 (− 0.7, 2.4)	0.288	1.2 (0.6, 1.8)	< 0.001	0.4 (0.1, 0.6)	0.013	0 (− 0.1, 0.2)	0.623
20s	4.0 (1.0, 7.1)	0.010	3.8 (0.8, 6.7)	0.012	1.0 (0.2, 1.7)	0.015	1.4 (0.7, 2.1)	< 0.001
30s	3.4 (1.5, 5.3)	< 0.001	0.5 (− 0.5, 1.5)	0.348	0.1 (− 1.2, 1.4)	0.900	0.8 (− 0.5, 2.0)	0.234
40s	− 1.6 (− 4.0, 0.7)	0.169	0.3 (− 2.1, 2.7)	0.801	− 0.7 (− 2.2, 0.8)	0.384	0 (− 1.2, 1.2)	0.958
50s	− 7.9 (− 11.5, − 4.2)	< 0.001	− 1.7 (− 3.3, − 0.1)	0.035	− 5.1 (− 7.2, − 2.9)	< 0.001	0.1 (− 1.1, 1.3)	0.876
60s	− 8.3 (− 13.1, − 3.5)	0.001	0.2 (− 4.2, 4.6)	0.938	− 5.2 (− 8.5, − 2.0)	0.002	− 1.3 (− 2.5, − 0.2)	0.018
70s	− 7.9 (− 18.4, 2.6)	0.142	− 13.1 (− 21.9, − 4.4)	0.003	− 12.1 (− 18.4, − 5.9)	< 0.001	− 4.1 (− 8.3, 0.1)	0.057
80s	− 62.6 (− 81.7, − 43.6)	< 0.001	− 40.9 (− 53.5, − 28.4)	< 0.001	− 27.0 (− 36.4, − 17.7)	< 0.001	− 12.4 (− 19.1, − 5.7)	< 0.001

Diff = Difference between observed 2020 case notification rate per 100,000 population and estimated 2020 case notification rate per 100,000 population. Positive values mean that observed rates were higher than estimated rates. Negative values mean that observed rates were lower than estimated rates; CI = confidential interval

Regarding new male cases, the observed CNRs in 2020 were higher than the estimated CNRs among younger males (Fig. 1E and Table 1). The observed CNRs were 20.9 and 24.8 per 100,000 population among ages 20–29 years and 30–39 years, respectively, while the estimated CNRs were 16.9 (95% confidence interval [CI] 13.0–20.7) and 21.5 (95% CI 19.1–23.8) per 100,000 population, respectively (P-value = 0.010 and < 0.001 (Table 1)). However, the observed CNRs in 2020 were lower than the estimated CNRs among older males (Fig. 1E and Table 1). Among males aged 50–59 years, 60–69 years, and ≥ 80 years, the observed CNRs of new cases per 100,000 population in 2020 were 50.6, 72.8, and 294.8, respectively, while the estimated CNRs per 100,000 population were 58.5 (95% CI 53.9–63.1), 81.1 (95% CI 75.1–87.0), and 357.5 (95% CI 333.7–381.3), respectively (P-value = < 0.001, 0.001, and < 0.001 (Table 1)). Similar trends were observed for new female cases (Fig. 1F and Table 1): The observed CNRs were higher than the estimated CNRs among younger females (0–29 years) and lower than the estimated CNRs among older females (≥ 70 years). The observed CNRs per 100,000 population among females aged 0–19 years and 20–29 years were 2.8 and 20.3, respectively, and the estimated CNRs per 100,000 population were 1.7 (95% CI 0.9–2.4) and 16.6 (95% CI 12.9–20.2), respectively (P-value = < 0.001, and 0.012 (Table 1)). The observed CNRs per 100,000 population among females aged 70–79 years and ≥ 80 years were 79.1 and 204.6, respectively, and estimated CNRs per 100,000 population were 92.2 (95% CI 81.3–103.1) and 245.5 (95% CI 229.8–261.2), respectively (P-value = 0.003 and < 0.001 (Table 1).

In males, regarding previously treated cases, the observed CNRs were not markedly lower than the estimated CNRs in younger age groups, but were lower than the estimated CNRs in older age groups (≥ 60 years) (Fig. 1G and Table 1). Among females, the observed CNR in the 20–29-years age group was 3.1 per 100,000 population, while the estimated CNR was 1.7 per 100,000 population (95% CI 0.9–2.6) (P-value = < 0.001 (Table 1)). In the ≥ 80 years group, the observed CNR was 35.5 per 100,000 population, while the estimated CNR was 47.9 per 100,000 population (95% CI 39.5–56.2) (P-value = < 0.001 (Table 1)).

## Discussion

Our study is consistent with previous studies that found that the observed TB CNR in 2020 was markedly lower than the expected CNR based on the past CNR trends [7, 8]. However, in our study, the magnitude of the difference between the observed and expected CNRs differed according to age. These results may be explained by a combination of positive and negative effects of COVID-19 control measures on TB, as proposed by McQuaid et al. [3] and Zumla et al. [5]: (1) reduced notification rate due to health service disruption; (2) increased transmission within households due to increased contact; (3) prevention of transmission in the community due to social distancing; and/or (4) increased TB diagnosis owing to the similarity of the respiratory symptoms to COVID-19.

First, we found the low TB CNR among adults aged ≥ 60 years. We speculate that this may be attributable to reduced access to essential health care services. In this age group, reactivation of latent TB infection predominates over recent infections, such that the effect of social distancing practices in the prevention of transmission may have been limited [9]. South Korea had a relatively low excess mortality during the COVID-19 pandemic, but the ratio of the other cause of deaths to direct COVID-19 deaths was the highest among 29 high-income countries, being fivefold higher [10]. The highest ratio may imply that essential health care services for other conditions including TB might have been compromised. However South Korea had successfully contained COVID-19 cases and mortality in the early stage of the pandemic [10]. Moreover, the utilization of emergency departments among the older was increased during the pandemic compared with the previous year (2018–2019), which may reflect delayed health utilization [11]. Particularly, older adults (≥ 65 years) may be more vulnerable to the disruption of essential health services than younger adults, provided that many older adults lack essential resources and the elderly poverty rate in South Korea is approximately 50%, the highest among OECD countries [12].

Second, we found that the observed TB CNRs were higher than the estimated CNRs among ages < 39 years. A Spanish study reported that the incidence of latent TB infection and active TB increased in child contacts due to frequent household contact [13]. We hypothesize that our finding may be related to increased transmission in households due to increased contact by social distancing and engagement in active health-seeking behavior after the onset of respiratory symptoms like COVID-19 symptoms. Further studies are needed to test this hypothesis.

Our results should be interpreted with caution, considering some key limitations. This study is a report from a single country with an intermediate TB burden, and our results do not directly support the expected impact of COVID-19 on TB as hypothesized in previous studies [3, 5]. Further, we were unable to conduct a multi-dimensional analysis using monthly data, considering the burden of the COVID-19 pandemic and changes in the TB CNR. The impact of COVID-19 control-measures on essential health care, such as TB care, is multifaceted. Moreover, the impact of COVID-19 on the healthcare system would be broader and not limited to TB care. Future studies should focus on specifically identifying the association between COVID-19 control measures and TB incidence based on individual-level clinical data. Moreover, the integration of TB programs with COVID-19 control measures such as systematic screening for respiratory symptoms should be considered as a matter of national policy.

## Data Availability

The datasets used and/or analyzed during the current study are available as a additional file.

## Abbreviations

CNR: Case notification rate
COVID-19: Coronavirus disease
TB: Tuberculosis
